# The Need for Original Investigation

**Published:** 1898-05

**Authors:** 


### Editorial.

### THE NEED FOR ORIGINAL INVESTIGATION.

The fundamental basis of all knowledge is absolute truth, and absolute truth can alone be found by seeking for it, not in imaginary ideals, but upon a positive substratum of fact. The search for the absolute in the unknown has been the work of the so-called scientific thought of the ages, and will continue until the secrets of life are more and more unfolded, each development adding to the intelligence and broader conception of mankind.

To each department of work in the world is given the elucidation of its special problems, and the responsibility rests with it to continue that labor unceasingly. To dentistry has been given the upbuilding of a profession, and it is needless to add that this has been reasonably well done, but because much has been gained it does not, necessarily, follow that all problems have been solved, or that we can rest content with the labors of the past.

Dentistry had its origin in mechanics, and while it possessed many cultivated men in its ranks, both in Europe and America, prior to the middle of the present century, it failed to merit the name of a profession. All the improvements made up to that time were of a mechanical order, hence the boundary of investigation was circumscribed, but exceedingly valuable, furnishing a foundation for future work of an important character. It was not until the formation of the first dental college by Chapin A. Harris in 1840 that the profession of dentistry can date its rise. From this period the work assumed a broader character and a more extended responsibility. It meant a call to the dental world to unification of effort, and, if it had no other signification, this was of vital importance. The effect upon the dental mind was not immediate, in fact for thirty years it was a question with many whether it would be possible to overcome the inertia of the mass, imbued, as it was, with the trade instinct rather than the scientific spirit.

It is due largely to the work of a persistent body of earnest men in Europe and this country that at last a true professional feeling was aroused, and with this came an increase of dental educational institutions everywhere, followed by restrictions of law, until the crudities of the past have been, if not entirely obliterated, at least forced to assume a subordinate part.

While all this is true and universally recognized, it is not equally appreciated that to continue this something more is needed than a mere passive acceptance of past work, as though all had been accomplished necessary for the well-being of our calling. It is feared that this spirit of inactivity has begun its deadly work in undermining the life of dentistry. While it is true that a few men are earnestly laboring in scientific explorations, these are in such a small minority that they are almost lost in the mass, and, it is feared, their work fails in the impress it deserves.

If this be not true, what means the rush for journals that condense in a few lines the work, it may be, of months, if it be not to cater to that indolence and vacuity of mind that refuses to give even the time required to read and study thoroughly the work of others? It means, if it means anything, that the dental profession has not time or disposition to think, much less to read or to act. There is nothing so discouraging to the true teacher than to find the student receiving listlessly the scientific side of his work, but will, as Dr. Peirce remarked in the last number of this journal, greedily ingather all that will aid him in his future financial operations. Dentistry will never be a true profession until the members thereof give to the accumulation of money the second place in their ambition. The cry is constantly heard, "We have no use for journals with labored scientific articles. Give us something that we can apply to every-day practice." The answer to this must be that the so-called scientific articles are the cream of thought and experience, and to miss which means the feeding upon the residuum, which, while it has a value, may be regarded as the pabulum for babes and not the food suitable for a vigorous and growing profession.

If there is one need more imperative than another in dentistry it is an effort to rouse the sluggish mentality to work, or at least to a more thorough conception of the necessity for that which does not pay in a financial sense. Until something better is presented than in much of our periodical literature, the outlook must assume pessimistic proportions. The journals of a profession rise to the dead level of those who read them, and can go no higher. Unfortunately, the journals of this country, with two or three exceptions, receive their impulse from trade, and whenever a profession levels down to that it cannot rise to a recognized scientific basis.

It is unfortunately true that our professional work is most exhausting, and individuals degenerate in it from sheer lack of moral courage to do more than the day's work requires, but some meet this difficulty, and others should follow if we are to maintain the respect of the thinking world.

It is a pleasure to recognize the fact that many of the younger men are imbued with the altruistic spirit of research, and while gratefully acknowledging this, it is hoped that more will repay that which they have received during their student years, for the dictum of Bacon, that "I hold every man a debtor to his profession," is as forceful to-day as when he wrote. Original investigation is the crying need of the present era. It is more than ever desirable to know what things are rather than what they ought to be in the imaginations of writers. Our literature is loaded down with pages upon subjects in which barrenness of original thought is but poorly compensated for by prolixity of words. It is a truism to say, every one has the germ of originality, and it is equally true that it simply requires an effort to bring it forth. The baleful dependence upon authority must be overcome if this original thought can see the light. "Take truth for authority and not authority for truth," is such a valuable precept that it should be engraven upon every mind. Seek the fountains for the source, and not the flowing rivers or the boundless oceans. It may be said, with some degree of truth, that this individualized seeking for light will come in time, but while this may be the ultimate result, it can never take place unless a broader example is set than exists at present. The cry is not now for more books, more periodicals, but for more readers, for more workers, and when this demand is filled then will dentistry be truly worthy of the name profession, which it is feared it does not deserve at present. We must grow out of the crudities of the past and rise worthy the age in which we are workers, for no one is justified in resting contentedly with the step gained, for another always lies before us for something higher.

